# Toxic Effect of Methyl-Thiophanate on *Bombyx mori* Based on Physiological and Transcriptomic Analysis

**DOI:** 10.3390/genes15101279

**Published:** 2024-09-29

**Authors:** Zhen He, Yang Fang, Fengchao Zhang, Yang Liu, Xiaoli Wen, Cui Yu, Xinkai Cheng, Dechen Li, Liang Huang, Hui Ai, Fan Wu

**Affiliations:** 1 Industrial Crops Institute, Hubei Academy of Agricultural Sciences, Wuhan 430064, China; zhenhe_nky@hbaas.com (Z.H.); yucui@hbaas.com (C.Y.); lidechen@hbaas.ac.cn (D.L.); hl820381079@126.com (L.H.); 2 Institute of Evolution & Marine Biodiversity, Ocean University of China, Qingdao 266100, China; fangyang@stu.ouc.edu.cn (Y.F.); 11210611009@stu.ouc.edu.cn (F.Z.); lysold112358@foxmail.com (Y.L.); chengxinkai@stu.ouc.edu.cn (X.C.); 3 College of Life Sciences, Central China Normal University, Wuhan 430079, China; chains72@163.com

**Keywords:** growth, oxidative stress, transcriptome, cytochrome P450

## Abstract

Background/Objectives: The utilization of methyl-thiophanate (MT) in vegetables and fruits is widespread due to its broad efficiency, yet its potential impact on silkworm growth remains uncertain. This study aims to examine the effects of MT on the growth of silkworms. Specifically, we assessed the weights of fifth-instar larvae that were fed mulberry leaves saturated with three concentrations (2.5, 5, and 10 mg/mL) of MT, as well as the weights of a control group. Methods: TEM was used to show the status of the silkworm midgut after MT supplementation. Oxidative stress was evaluated in the presence of MT. Furthermore, a transcriptomic sequencing experiment was conducted to investigate the mechanism through which the development of silkworms is induced by MT. Results: Our findings indicate that the supplementation of MT hindered larval growth compared to the control group, suggesting a toxic effect of MT on silkworms. The transmission electron microscopy (TEM) results show that MT supplementation induced autophagy in the silkworm midgut. MT was also found to induce oxidative stress in silkworms through the activation of reactive oxygen (ROS), superoxide dismutase (SOD), catalase (CAT), and peroxidase (POD) activities. Subsequent transcriptomic analysis revealed 1265 significantly differentially expressed genes (DEGs) in response to MT. Kyoto Encyclopedia of Genes and Genomes (KEGG) analysis indicated that these DEGs were associated with antioxidant defense, detoxification processes, lysosome biogenesis, and metabolic pathways. Conclusions: These findings suggest that MT toxicity in silkworm larvae is mediated through the induction of oxidative stress and alterations in metabolism. This study contributes to our understanding of the impacts of MT exposure on silkworms and provides insights into potential pesticides for use in mulberry gardens.

## 1. Introduction

The silkworm (*Bombyx mori* L.) is a crucial economic insect in China, contributing significantly to the country’s economic growth. Due to its short life cycle and easy feeding, silkworms have been utilized as model organisms for toxicological assessment, developmental biology studies, genetic investigations, molecular biology research, and medical research [1,2,3,4]. Mulberry leaves serve as the primary natural food for silkworms; additionally, the mulberry tree (*Morus alba* L.) is renowned for its nutritious and delicious fruit [5,6]. In recent years, mulberry trees have gained recognition as viable food sources for animals, primarily due to their palatability, nutrient-rich composition, abundance of vitamins, minerals, and various natural active compounds [7,8]. This has led to the mulberry tree’s emergence as a versatile economic species, with the mulberry industry serving as a prominent example of the diversified development within sericulture. In the practice of sericulture, the cultivation of mulberry gardens is essential for optimizing the benefits derived from mulberry trees, as they serve as a source of leaves for feeding silkworms, fruits for consumption, and fodder for animals. Given that mulberry trees vary in their utility for these purposes, strategic development of mulberry gardens is crucial for maximizing productivity. The harvesting of mulberry fruits typically occurs in spring, while silkworm breeding take places in both spring and autumn to ensure optimal yields of silkworm cocoons and mulberry fruits.

Pesticides are integral to agricultural production in prominent agricultural nations, resulting in the frequent contamination of fruits and vegetables with pesticide residues [9,10,11]. Methyl-thiophanate (MT), a thioallophanate compound, is widely utilized as a fungicide to combat crucial fungal diseases in crops, owing to its superior efficacy compared to other commonly employed fungicides [12]. MT is commonly utilized in Chinese agriculture for various crops, including strawberries [13,14], and mulberries [15], bananas [16], and papayas [17] due to its notable efficacy. Upon metabolism in humans and other mammals, MT is transformed into benzimidazole compounds, such as carbendazim [18]. Numerous investigations have demonstrated the toxic effect of MT on zebrafish, rats, and various other organisms, thereby posing potential toxicological hazards to the general population [18,19,20,21,22]. For example, exposure to MT resulted in developmental toxicity in zebrafish, manifested as impaired hatchability, reduced heart rates, restricted spontaneous locomotion, and body length reduction [20]. Furthermore, Che (2023) et al. discovered that MT triggers notochord toxicity through the activation of the PI3K-mTOR pathway [20]. In rats, the toxicity induced by MT can result in significant production of reactive oxygen species (ROS), ultimately causing severe genotoxicity [18].

In a mulberry garden, there are different kinds of mulberry species. For example, mulberry trees for fruits and mulberry trees for cultivating silkworms. MT is commonly used in mulberry trees (for fruits) for controlling fungi. MT may contaminate the leaves for feeding silkworms. This study aimed to assess the impact of MT on silkworm growth in mulberry gardens and the mechanism through which the development of silkworms is induced by MT. These findings may be utilized to assess the impact of MT on silkworms, and they offer scholarly insights into the application of MT in mulberry garden cultivation.

## 2. Materials and Methods

### 2.1. Silkworm Strains and Rearing

Freshly molted silkworm larvae (Chufeng × Hanyun strains) were procured from the Industrial Crops Institute, Hubei Academy of Agricultural Sciences, located in Wuhan, China. The silkworm undergoes four molting cycles, during which it sheds its skin and experiences incremental growth. The period between each molting cycle is referred to as an “instar”. Consequently, a silkworm that has molted four times is considered to be in its fifth instar. All fifth-instar larvae of similar size were raised under a 12 h light/dark cycle at a consistent temperature of 25 ± 1 °C, maintaining a relative humidity level of 70–75%, as previously described [1,23].

### 2.2. Methyl-Thiophanate Content Determination

Fresh and healthy mulberry leaves were harvested from the mulberry garden and immersed in solutions containing three concentrations of MT (2.5, 5, and 10 mg/mL) for 5–10 min to ensure uniform coating. The leaves were then air-dried at room temperature for 1 h. The weights of the fresh and dried leaves were measured after oven-drying at 70 °C. Approximately 25 g of dried leaves was placed in a 100 mL centrifuge tube with 25 mL of acetonitrile. After homogenization, 15 g of anhydrous magnesium sulfate was added to the tube, which was then vigorously shaken for 1 min. The supernatant obtained through centrifugation at 2000× *g* for 30 min was subjected to further processing. Specifically, 1 mL of the supernatant was combined with 200 mg of anhydrous magnesium sulfate and 50 mg of primary secondary amine (PSA) in a 2 mL centrifuge tube. Additionally, 0.5 mL of the supernatant was mixed with 0.5 mL of an ion pairing agent, prepared by mixing 200 mL of ultrapure water with 7 mL of phosphoric acid, 1 g of sodium sulfonate, and 10 mL of triethylamine, and then diluting to a final volume of 1000 mL. The resulting filtrate was subjected to testing after passing through a 0.45 µm filter membrane. The determination of MT content in mulberry leaves was carried out using high-performance liquid chromatography (HPLC) at Hubei Yile Technology Co., Ltd. (Wuhan, China). The detection limitation of MT was 0.09 mg/kg.

### 2.3. Methyl-Thiophanate Treatment

MT powder was diluted with ultrapure water to create various concentrations. Healthy mulberry leaves were collected from the garden and immersed in MT solutions of 2.5, 5, and 10 mg/mL for 5–10 min to be evenly coated with MT. The leaves were then air-dried at room temperature for 1 h. The control groups were provided with mulberry leaves soaked in ultrapure water, as previously described [1,23].

In all feeding experiments, fifth-instar larvae were utilized post-fourth molt. Each biological replicate compromised 60 healthy larvae of uniform age and size, with 3 biological replicates per group (*n* = 180). Various concentrations of MT were administered to the larvae in each group every eight hours from 0600 to 2200: 150 g of mulberry leaves was provided on days 1 and 2, 180 g on days 3 and 4, and 150 g on days 5 and 6, as previously described [1,23]. Prior to subsequent feedings, any remaining mulberry leaves and deceased larvae were consistently removed, measured, and documented. The weight and number of deceased larvae were documented prior to the initial feeding each day.

### 2.4. Transmission Electron Microscopy (TEM)

Four fifth-instar larvae were obtained from the MT-treated (10 mg/mL) and control groups on day 4. The midguts were isolated from the larvae anesthetized with CO_2_ and immediately fixed in 2.5% glutaraldehyde (Solarbio, Beijing, China) at 4 °C overnight. The fixed testes were then rinsed in 0.2 mol/L phosphate buffer (pH 7.4) and post-fixed in 1% osmium tetroxide (OsO4) for one hour. Following the double fixation process, the samples underwent dehydration through a graded ethanol series and were subsequently embedded in Araldite (EMbed 812, Hatfield, PA, USA). Ultrathin sections were prepared using the Leica Ultracut 6b microtome (Leica, Wetzlar, Germany), subsequently stained with uranyl acetate and lead citrate, and imaged utilizing a JSM-2100 KV transmission electron microscope (JEOL, Tokyo, Japan).

### 2.5. Reactive Oxygen Species (ROS) Assay

The level of ROS was assessed by the interaction between the hydroxyl radical and the oxidation-sensitive probe, 2, 7-dichlorofluorescein diacetate (DCFH-DA) (Applygen, Beijing, China). Following a reaction with ROS, DCFH-DA is converted to DCFH, which can be measured using a FLx 800 fluorescence reader (Bio-Tek, Winooski, VT, USA). Four fifth-instar larvae from the fourth day from both the treated group (10 mg/mL) and the control group were pulverized with liquid nitrogen and combined with 500 µL of 1 × phosphate-buffered saline (PBS). Following homogenization for 3 min and centrifugation at 2800× *g* for 3 min, a 30 µL supernatant was diluted to 100 µL with PBS. Subsequently, the diluted sample was combined with 100 µL DCFH-DA (30 µmol/L) and transferred to a microplate well. After an incubation period of 30 min at 37 °C in complete darkness, the level of ROS was measured using a fluorescence reader set at 485 nm and 520 nm. The ROS level was quantified and expressed as units per milligram of protein (U/mgprot).

### 2.6. Measurement of Superoxide Dismutase (SOD) Activity, Catalase (CAT) Activity, and Peroxidase (POD) Activity

The activities of SOD, CAT, and POD were assessed using detection kits purchased from Nanjing Jiancheng Bioengineering Institute (Nanjing, China) following the manufacturer’s instructions. Briefly, four fifth-instar larvae on the fourth day were pulverized using liquid nitrogen, and then suspended in 500 µL of PBS from both the treated group (10 mg/mL) and the control group. The supernatant was obtained after homogenization and centrifugation at 8000× *g* for 10 min at 4 °C.

To assess the SOD activity, 18 µL of the supernatant was combined with 48 µL of PBS, 12 µL of xanthine oxidase, 36 µL of 0.037 U/L, 1.25 mmol/L nitrolated tetrazolium blue, 6 µL of xanthine, and 68 µL of ddH_2_O in a 96-well microplate. Following an incubation period at 37 °C for 30 min, the SOD activity was measured using a fluorescence reader set at 560 nm. The SOD activity was quantified as U/mgprot.

In the context of a CAT assessment, 20 µL of the supernatant was combined with 100 µL of 20 µmol/mL H_2_O_2_ and subsequently incubated for 10 min at room temperature. Following the addition of 180 µL of 50 mmol/L ammonium molybdate tetrahydrate, the CAT activity was quantified utilizing a fluorescence reader set to operate at a wavelength of 405 nm. The resulting CAT activity was reported as U/mgprot.

In the context of POD activity, a working solution was prepared consisting of 100 mmol/L PBS (pH = 6.0), guaiacol, H_2_O_2_, and ddH_2_O at a ratio of 104:26:27:37. Then, 20 µL of the supernatant was combined with 204 µL of the working solution in a 96-well microplate. The POD content was quantified using a fluorescence reader to measure absorbance values at 470 nm at 30 s 5 min and 30 s. The resulting POD content was then expressed as U/mgprot.

### 2.7. RNA Preparation and Sequencing

RNA extraction and sequencing were conducted by BioMarker (Beijing, China) on four fifth-instar larvae obtained from the MT-treated (10 mg/mL) and control groups on day 4. The total RNA was extracted using TRIzol^®^ Reagent (Thermo Fisher Scientific, Waltham, MA, USA) according to the manufacturer’s protocol. The concentration of the total RNA was measured using a Nanodrop ND-2000 (Thermo Scientific), and the integrity was assessed using an Agilent Bioanalyzer 4150 system (Applied Biosystem, Foster City, CA, USA). Paired-end libraries were prepared following the protocol provided by the ABclonal RNA-seq Lib Prep Kit (ABclonal, Wuhan, China). The mRNA was isolated using oligo-dT magnetic beads, the fragmented mRNA was converted to cDNA with SuperScript II reverse transcriptase, indexed Illumina adapters were attached, and limited-cycle PCR was applied. The resulting PCR products were purified with the AMPure XP system, and the library quality was assessed using the Agilent Bioanalyzer 4150. Paired-end 150 bp reads were obtained using either the Illumina Novaseq 6000 (San Diego, CA, USA) or MGISEQ-T7 (Shenzhen, China).

### 2.8. Analysis of Transcriptome Data

The reference genome information used in this project was Vitis_vinifera.12X.dna.toplevel.fa. Initially, the raw data underwent a filtration process to produce high-quality sequences (clean data), which were subsequently aligned to the reference genome of silkworm by HISAT 2 (Hierarchical Graph FM index for Sequence Alignment). We employed HTSeq for statistical alignment to obtain the read count values for each gene, representing the original gene expression levels. The read counts demonstrated a positive correlation with the true expression levels of the genes, as well as with the gene length and sequencing depth. To facilitate the comparability of the gene expression levels across different genes and samples, we standardized the expression levels using fragments per kilobase of transcript per million mapped reads (FPKM). In paired-end sequencing, each fragment is represented by two reads, and the FPKM (fragments per kilobase of transcript per million mapped reads) metric quantifies the number of fragments for which both reads align to the same transcript. Within the reference transcriptome, it is generally accepted that genes with an FPKM value > 1 are considered to be expressed. Differentially expressed genes (DEGs) were selected based on a log_2_(fold change) >1 and a *q*-value < 0.05 with three biological replicates by DESeq (differential expression analysis for sequence data 2). The false discovery rate (FDR) < 0.05 was applied to control the false positive rate. Subsequently, KEGG pathway analysis was performed to assess the statistical enrichment of DEGs using KOBAS software (v2.0) [24]. Both groups yielded a total of 3.82–4.16 million and 3.88–4.34 million clean reads (Table 1), with at least 93.18% of clean reads exhibiting Phred-like quality scores at the Q30 level (an error probability of 0.001).

### 2.9. Reverse Transcription Quantitative Transcriptase–Polymerase Chain Reaction (RT-qPCR)

Four fifth-instar larvae of the whole body from the fourth day from both the MT-treated group (10 mg/mL) and the control group were collected. The total RNA was extracted using TRIzol (Thermo Fisher Scientific) according to the manufacturer’s protocol. Briefly, first-strand cDNA was synthesized from approximately 1–2 μg of the total RNA using reverse transcriptase (EasyScript, TransGen, Beijing, China) and oligo dT18 primer at 42 °C for 60 min. Specific primers were designed based on sequences obtained from the sequencing results (Table S1). An RT-qPCR assay was conducted to assess the transcription level of specific genes in comparison to the housekeeping gene *Actin 3*. The gene expression analysis was carried out using a CFX Connect^TM^ Real-Time System (BioRad, Hercules, CA, USA) and a SYBR Green qPCR Mix (TransGen). Each reaction consisted of 20 μL volume, comprising 10 μL of 2 × SYBR premix, 0.5 μL each of forward and reverse primers (10 μM), 2 μL of cDNA template diluted 20-fold with deionized H_2_O, and deionized H_2_O to reach a final volume of 20 μL. The RT-qPCR protocol involved an initial denaturation step at 95 °C for 3 min, followed by 40 cycles of amplification at 95 °C for 10 s, 58 °C for 20 s, and 72 °C for 20 s. Subsequently, a melting curve analysis was conducted over a temperature range of 55 °C to 98 °C. The 2^−ΔΔCT^ method was employed for quantification of relative transcriptional changes. The experimental replicates included three biological replicates and two technical replicates per sample, as described in previous studies [25,26].

### 2.10. Sequence Alignment

Homologous sequences exhibiting greater than 60% amino acid sequence similarity to the cytochrome P450 protein were retrieved from the NCBI database. These sequences were subsequently analyzed using the SignalP online software 4.1 (SignalP 4.1-DTU Health Tech-Bioinformatic Services) to predict the signal peptides. Based on the prediction results, the signal peptides were excised from each sequence. The sequences were then renamed and aligned using DNAMAN software 6.0. The alignment results were exported as images for further analysis.

### 2.11. Motif Analysis

The cytochrome P450 protein sequences, excluding the signal peptides, were incorporated with homologous sequences into FASTA format files. Motif analysis of the cytochrome P450 protein sequences and their homologous counterparts was conducted utilizing the MEME online software 5.5.7 (meme-suit.org/meme/index.html, accessed on 16 August 2024). The results were visualized through the MEME HTML output. We downloaded the motif analysis results in XML format and exported the motif location map utilizing TBtools software (v2.119). Subsequently, based on the analysis results obtained from the MEME website, the amino acid residues preceding the first conserved motif of each sequence were excised. The sequences were then aligned using DNAMAN to standardize their lengths. The processed sequences were compiled into FASTA format documents, and a sequence identity map was generated using the WEBLOGO online software 3 (weblogo.berkeley.edu, accessed on 16 August 2024). Moreover, to enhance the analysis of conserved motifs, the functional domains of cytochrome P450 proteins can be predicted using the CD-Search tool available on the NCBI website (ncbi.nlm.nih.gov, accessed on 16 August 2024).

### 2.12. Statistical Analysis

A two-tailed Student’s *t*-test was conducted using GraphPad Prism 8.0 for all experiments, including the larval growth experiment, to compare the experimental groups and the control group. Statistical significance was determined as a *p*-value of less than 0.05.

## 3. Results

### 3.1. Content of Methyl-Thiophanate in Mulberry Leaves

**Figure 1:**
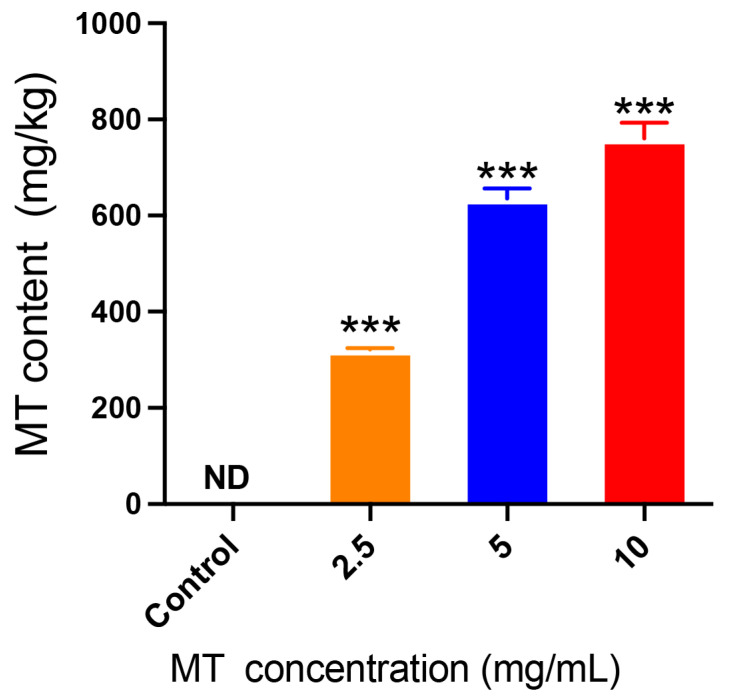
Content of MT in mulberry leaves. Data are presented as means ± SDs (*n* = 3). ***: *p* < 0.001. ND: Not detected.

The presence of MT in mulberry leaves serves as the primary source of MT transfer to the *B. mori* population. Initially, this study involved assessing the MT content in mulberry leaves exposed to varying concentrations of MT solutions. The results indicate that no MT was present in the control group’s mulberry leaves (Figure 1), with significantly lower concentrations observed compared to those treated with MT solutions. Furthermore, the concentration of MT in the mulberry leaves exhibited a proportional corresponding to the concentration of the treated MT solution (Figure 1). The concentration of MT in leaves reached 760.9 ± 18.49 mg/kg following treatment with a 10 mg/mL solution for 5–10 min.

### 3.2. Effects of Methyl-Thiophanate on Larval Development

**Figure 2:**
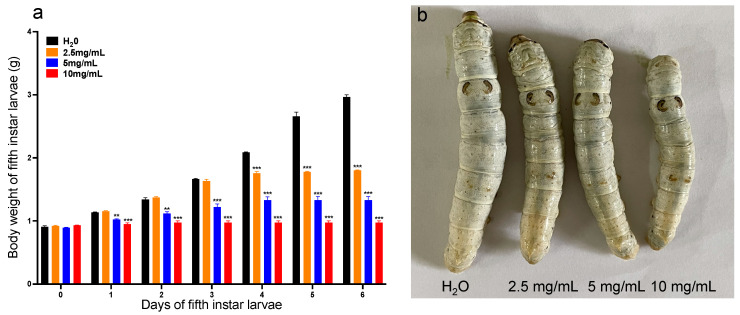
(**a**) Daily body weights of fifth-instar larvae. The data are presented as the means ± SDs (*n* = 3). **: *p* < 0.01; ***: *p* < 0.001. (**b**) The morphology of *Bombyx mori* larvae on the fourth day of the fifth instar stage after MT treatment.

The impact of MT exposure on the growth of silkworm larvae was examined, with a focus on changes in the larval body weight as a key indicator of development. Daily weight measurements of fifth-instar larvae were taken after supplementation with different concentrations of MT beginning on the first day of the fifth instar stage (Figure 2a). Prior to treatment, there were no significant differences in the average weights between the experimental and control groups (Figure 2a). However, supplementation with 5 and 10 mg/mL MT resulted in significant weight loss compared to the control group from days 1 to 6 (Figure 2a). In contrast, supplementation with 2.5 mg/mL MT showed no difference in the weight for the first 3 days, but a significant decrease from day 4 to 6 (Figure 2a). Additionally, it was observed that the larval size on the fourth day of the fifth-instar decreased with increasing concentrations of MT (Figure 2b).

### 3.3. Methyl-Thiophanate Supplementation Induced Autophagy

**Figure 3:**
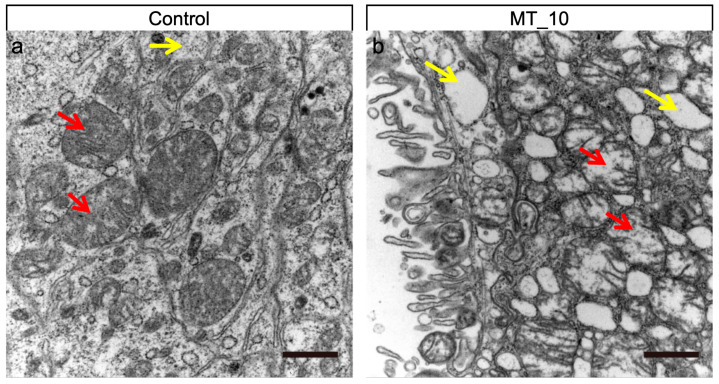
The general morphology of *Bombyx mori* larvae midgut on the fourth day of the fifth instar stage. Scale bar: 1 μm (**a**,**b**).

To evaluate the impact of MT on the midgut structure, TEM was employed. In the control group, intracellular mitochondrial morphology remained intact, with a clearly visible and intact mitochondrial inner membrane (Figure 3a, red arrows), and normal cytoplasmic autophagosomes (Figure 3a, yellow arrow). Conversely, the treatment group exhibited severe organic lesions in the midgut cells, disordered mitochondrial morphology (Figure 3b, red arrows), and stretched mitochondrial inner membranes. Post-treatment, the midgut cells demonstrated significant inflammation, accompanied by a substantial increase in autophagic vesicles (Figure 3b, yellow arrows).

### 3.4. Methyl-Thiophanate Supplementation Activated Larval Oxidative Stress

**Figure 4:**
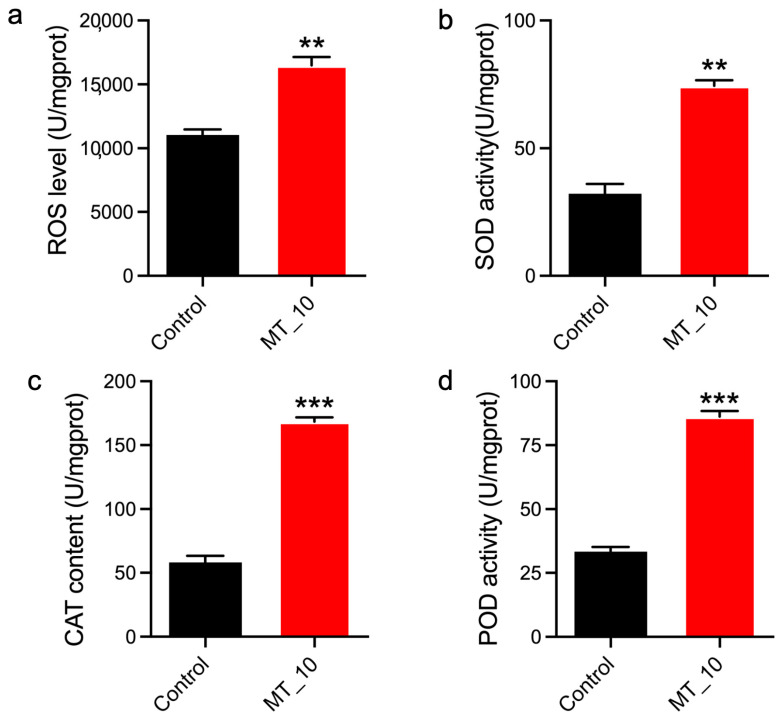
MT supplementation induced oxidative stress in silkworm larvae. (**a**) ROS levels were significantly increased by MT. (**b**) SOD activities were significantly increased by MT. (**c**) CAT activities were significantly increased by MT. (**d**) POD activities were significantly increased by MT. The data are presented as the means ± SDs (*n* = 3). **: *p* < 0.01 ***: *p* < 0.001. MT_10: MT-treated group (10 mg/mL).

To assess the potential involvement of MT challenge in oxidative stress, the levels of ROS, SOD, CAT, and POD were measured in the fifth-instar larvae exposed to MT. The supplementation of MT resulted in a significant increase in ROS levels, SOD activities, CAT activities, and POD activities (Figure 4).

### 3.5. Transcriptome Sequence and Assembly

This study analyzed the mRNA profiles of the MT-treated group (10 mg/mL, MT_10) and the control group to investigate the impact of MT on silkworm growth and development. The GC contents in the groups ranged from 45.93% to 47.54% and from 46.13% to 46.69%, respectively (Table 1). The alignment efficiency of clean reads to the *B. mori* reference genome for each sample varied between 74.70% and 92.59%, with less than 12.50% of the clean reads from the two groups exhibiting multiple alignment locations on the reference genome (Table 1). These findings suggest that the sequencing quality was notably high, facilitating further analysis.

### 3.6. Identification and Analysis of DEGs

**Figure 5:**
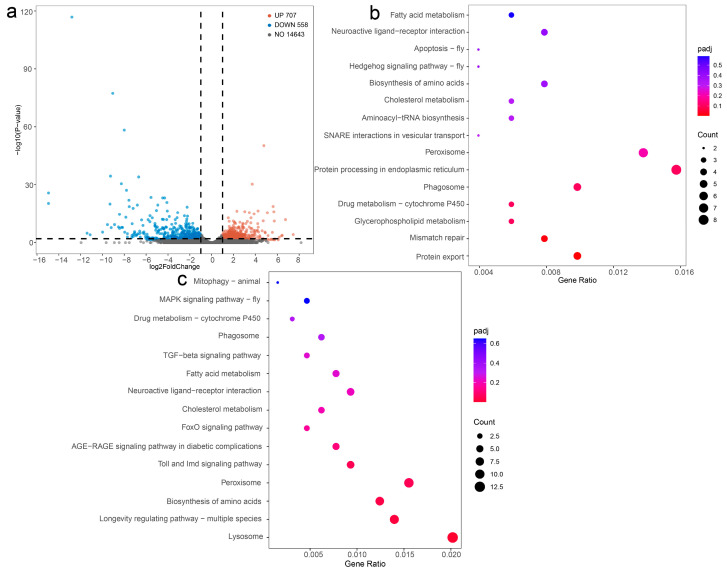
Volcano plot and KEGG pathway of differentially expressed genes of fifth-instar *Bombyx mori* larvae following MT treatment. (**a**) Volcano plot of differentially expressed genes of fifth-instar *Bombyx mori* larvae following MT treatment. The Y-axis represents the -log10 significance. The X-axis represents the log_2_ (fold change). The red dots represent upregulated genes, the green dots represent downregulated genes, and the blue dots represent unchanged genes. (**b**) The 15 most enriched KEGG pathways based on upregulated genes in the *Bombyx mori* transcriptome induced by MT. (**c**) The 15 most enriched KEGG pathways based on downregulated genes in the *Bombyx mori* transcriptome induced by MT.

In the fifth-instar larvae of *B. mori* induced by MT, a total of 1265 DEGs were identified, with 707 upregulated and 558 downregulated, meeting the criteria of a functional *q*-value < 0.05 and log_2_ (fold change) > 1 (Figure 5a and Table S2). Subsequent KEGG pathway analysis was conducted to elucidate the potential functions of the DEGs, revealing enrichment in 44 pathways with 1403 DEGs annotated. The results show that “Lysosome” had the largest number of DEGs in the upregulated KEGG pathway (Figure 5b, Table S3). Furthermore, the enrichment of “Longevity regulating pathway-multiple species”, “Biosynthesis of amino acids”, and “Peroxisome” in the analysis (Figure 5b and Table S3) suggests that MT primarily impacts the metabolic pathways of silkworm larvae. In the downregulated DEGs, the results show that “Protein export” had the largest number of DEGs in the upregulated KEGG pathway (Figure 5c, Table S4).

### 3.7. DEGs Involved in Antioxidation, Detoxification, Metabolism, and Autophagy

**Figure 6:**
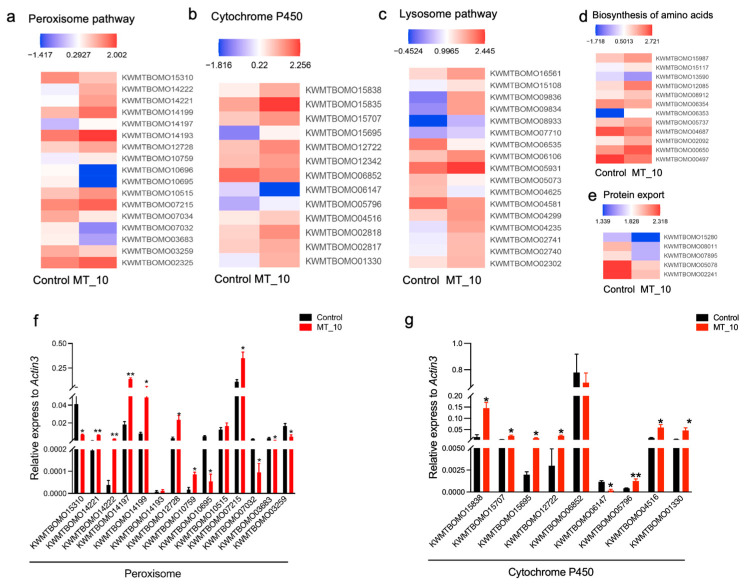
Heatmaps of antioxidation, detoxification, and lysosome gene expression in the present study. (**a**) The expression level of the peroxisome pathway. (**b**) The expression level of the cytochrome P450 pathway. (**c**) The expression level of the lysosome pathway. (**d**) The expression levels of genes related to the amino acid biosynthesis pathway. (**e**) The expression levels of genes involved in protein export. The color scale is shown at the upper left, ranging from the lowest (blue) to the highest (red) log10 (expression) value. (**f**) Quantitative reverse transcriptase–polymerase chain reaction validation of differentially expressed genes related to the peroxisome pathway in silkworms induced by MT. (**g**) Reverse transcription quantitative transcriptase–polymerase chain reaction (RT-qPCR) validation of differentially expressed genes related to the peroxisome pathway in silkworms induced by MT. The data are presented as the means ± SDs (*n* = 3). *: *p* < 0.05 **: *p* < 0.01. The heatmaps represent the log_10_ (averaged values of 3 replicates of FPKM).

This study identified several genes involved in antioxidant and detoxification processes (Figure 6), with a total of 17 genes in the peroxisome pathway associated with antioxidant systems. Within this pathway, five fatty acyl-CoA reductase genes (*KWMTBOMO14222*, *KWMTBOMO14221*, *KWMTBOMO14199*, *KWMTBOMO14197*, and *KWMTBOMO14193*), one aspartate oxidase gene (*KWMTBOMO12728*), one acetaldehyde oxidase gene (*KWMTBOMO10759*), one alkylglycerone-phosphate synthase gene (*KWM-TBOMO10515*), one xanthine dehydrogenase gene (*KWMTBOMO07215*), and one long-chain-fatty-acid-CoA ligase 4 gene (*KWMTBOMO02325*) were found to be upregulated, while two xanthine dehydrogenase genes (*KWMTBOMO10696* and *KWMTBOMO10695*), two fatty acyl-CoA reductase genes (*KWMTBOMO07034* and *KWMTBOMO07032*), one aspartate oxidase gene (*KWMTBOMO03683*), and one peroxisomal biogenesis factor 11 gene (*KWMTBOMO03259*) were downregulated (Figure 5a).

In the context of detoxification, eleven cytochrome P450 (CYPs) genes (*KWMTBOMO15838*, *KWMTBOMO15835*, *KWMTBOMO15707*, *KWMTBOMO15695*, *KWMTBOMO12722*, *KWMTBOMO12342, KWMTBOMO05796*, *KWMTBOMO04516, KWMTBOMO02818*, *KWMTBOMO02817*, *and KWMTBOMO01330)* were found to be upregulated, while two genes (KWMTBOMO06852 and KWMTBOMO06147) were downregulated, as depicted in Figure 5b.

Furthermore, a total of 13 genes (*KWMTBOMO16561*, *KWMTBOMO15108*, *KWMTBOMO09836*, *KWMTBOMO09834*, *KWMTBOMO08933*, *KWMTBOMO07710, KWMTBOMO06106, KWMTBOMO05931*, *KWMTBOMO04299, KWMTBOMO04235, KWMTBOMO02741*, *KWMTBOMO02740*, and *KWMTBOMO2302)* associated with the lysosome pathway exhibited upregulation, while 4 genes (*KWMTBOMO06535*, *KWMTBOMO05073*, *KWMTBOMO04625*, and *KWMTBOMO04581*) displayed downregulation (Figure 6c). In terms of the amino acid biosynthesis pathway, 10 genes showed upregulation, with 1 gene demonstrating downregulation (Figure 6d). Moreover, all genes related to protein export were downregulated (Figure 6e).

In order to validate the findings identified in the transcriptome sequencing analysis, a subset of genes associated with the peroxisome pathway and CYP pathway were chosen for assessment of their relative expression levels using RT-qPCR. The results indicate that 8 out of 14 genes were upregulated, while 3 out of 14 genes were downregulated within the peroxisome pathway (Figure 6f). Similarly, within the cytochrome P450 pathway, 7 out of 9 genes were upregulated, and 1 out of 9 genes was downregulated (Figure 6g).

### 3.8. Motif Analysis of the Cytochrome P450 Proteins and Their Homologous Sequences

To conduct a more in-depth analysis of the role of CYPs in response to MT, the three genes exhibiting the most significant fold changes by RT-qPCR were selected for bioinformatics analysis (*KWMTBOMO15838*, *KWMTBOMO12722*, and *KWMTBOMO01330*). Following the prediction of signal peptides in the proteins of these silkworm CYPs and the corresponding amino acid sequences in other species using SignalP-4.1 online software, we excised the signal peptides and subsequently conducted homologous sequence alignment utilizing DANMAN software 6.0. The results show that the sequence similarity of the silkworm BmorCYP4C1 (KWMTBOMO15838) protein with its homologs in other species was determined to be 77.06% (File S1). Similarly, the BmorCYP4C3 (KWMTBOMO01330) protein of the silkworm exhibited a sequence similarity of 77.34% with its counterparts in other species (File S3). The BmorCYP6B1 (KWMTBOMO12722) protein of the silkworm showed a sequence similarity of 69.46% when compared to its homologous sequences in other species (File S2). These results suggest that the three silkworm CYPs exhibit a high degree of similarity in their amino acid residue compositions when compared to the CYPs retrieved from other species.

**Figure 7:**
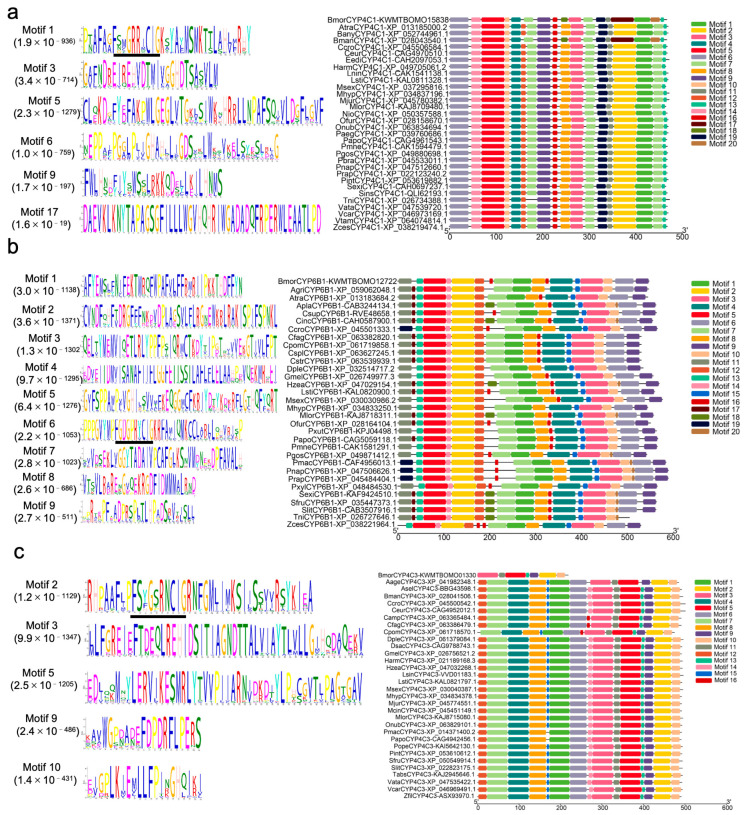
Motif analysis of three CYPs and homologous sequences. (**a**) Motif analysis of BmorCYP4C1 (KWMTBOMO15838) and homologous sequences. (**b**) Motif analysis of BmorCYP6B1 (KWMTBOMO12722) and homologous sequences. (**c**) Motif analysis of BmorCYP4C3 (KWMTBOMO01330) and homologous sequences. Black lines indicate heme-binding domain motif pattern (FXXGXRXCXG). Bmor: *Bombyx mori*, Atra: *Amyelois transitella*, Bany: *Bicyclus anynana*, Bman: *Bombyx mandarina*, Ccro: *Colias croceus*, Ceur: *Colias eurytheme*, Eedi: *Euphydryas editha*, *Harm: Helicoverpa armigera*, Lnin: *Leptosia nina*, Lsti: *Loxostege sticticalis*, Msex: *Manduca sexta*, Mhyp: *Maniola hyperantus*, Mjur: *Maniola jurtina*, Mlor: *Mythimna loreyi*, Nio: *Nymphalis io*, Ofur: *Ostrinia furnacalis*, Onub: *Ostrinia nubilalis*, Paeg: *Pararge aegeria*, Papo: *Parnassius apollo*, Pmne: *Parnassius mnemosyne*, Pgos: *Pectinophora gossypiella*, Pbra: *Pieris brassicae*, Pnap: *Pieris napi*, Pint: Plodia interpunctella, Sexi: *Spodoptera exigua*, Sins: *Streltzoviella insularis*, Tin: *Trichoplusia ni*, Vata: Vanessa atalanta, Vcar: *Vanessa cardui*, Vtam: *Vanessa tameamea*, Zces: *Zerene cesonia*, Agri: *Achroia grisella*, Apla: *Arctia plantaginis*, Csup: *Chilo suppressalis*, Cinc: *Chrysodeixis includens*, Cfag: *Cydia fagiglandana*, Cpom: *Cydia pomonella*, Cspl: *Cydia spendana*, Cstr: *Cydia strobilella*, Dple: *Danaus plexippus*, Gmel: *Galleria mellonella*, Hzea: *Helicoverpa zea*, Lsti: *Loxostege sticticalis*, Pxut: *Papilio xuthus*, Pmac: *Pieris macdunnoughi*, Prap: *Pieris rapae*, Pxyl: *Plutella xylpstella*, Sfru: *Spodoptera frugiperda*, Slit: *Spodoptera littoralis*, Aage: *Aricia, agestis*, Asel: *Ascotis selenaria*, Camp: *Cydia amplana*, Dsac: *Diatraea saccharalis*, Lsin: *Leptidea sinapis*, Mcin: *Melitaea cinxia*, Pope: *Phthorimaea operculella*, Tabs: *Tuta absoluta*, Zfil: *Zygaena filipendulae*.

The conserved motifs of Bmor4C1 proteins and their homologous sequences were analyzed using the MEME Suite. This analysis identified 20 conserved motifs across the 32 sequences, designated as Motif 1 through Motif 20 (Figure 7a). Notably, Motifs 5 and 17, were the longest, each consisting of 50 amino acids. Interestingly, Motif 17 is only present in silkworms (BmorCYP4C1 and BmanCYP4C1), suggesting its role in silkworms. Similarly, the analysis identified 20 conserved motifs across the 32 sequences, also designated as Motif 1 through Motif 20 (Figure 7b). Among these, Motif 2 through Motif 5 were the longest, each comprising 50 amino acids. Specifically for BmorCYP4C3, 16 conserved motifs were identified across the 32 sequences, designated as Motif 1 through Motif 16. Among these, Motif 3 and Motif 5 were the longest, each comprising 50 amino acids (Figure 7c). Insect CYP4 and CYP6 genes families have a relatively conserved heme-binding domain motif pattern (FXXGXRXCXG) and contain conserved cysteine residues (Figure 7). BmorCYP4C1 (Motif 1), BmorCYP6B1 (Motif 6), and BmorCYP4C3 (Motif 2) also formed the fifth axial ligand of heme iron, which has been reported to be related to physiological functions, such as insecticide resistance, detoxification mechanism, and odor degradation. Future research will focus on investigating the role of these three CYPs in silkworm detoxification and resistance against oxidative stress induced by MT.

## 4. Discussion

As the economy has evolved, the traditional function of mulberry gardens as silkworm-rearing facilities has expanded to include the cultivation of mulberry fruits. These fruits are highly valued for their abundance of vitamins and medicinal properties, which can be utilized in the production of beverages, fruit wines, and condiments that promote human health. In addition, mulberries are known for their palatable taste, nutrient-rich composition, diverse mineral content, and presence of various natural bioactive compounds, making them an ideal dietary supplement for animals [7,8]. MT has been utilized in the cultivation of mulberry trees to mitigate the impact of pests and diseases, thereby enhancing fruit yield and quality [15]. Given the susceptibility of silkworms to environmental factors, including their limited tolerance to pesticides, the potential effects of MT as a fungicide on silkworms have become a focal point of research in the advancement of mulberry gardens, for both fruit production and as a food source for silkworms. In the present study, the effects of MT on the growth of silkworm were examined. Fifth-instar silkworm larvae were exposed to MT concentrations of 2.5, 5, and 10 mg/m, resulting in inhibited larval growth, as demonstrated in Figure 2. These findings are in accordance with prior research indicating the toxic effects of MT on zebrafish and rats [18,19,20,21,22].

Oxidative stress is identified as a key factor in the toxicity induced by agrochemicals [27,28,29,30,31,32]. MT can lead to the accumulation of ROS, which in turn can cause genotoxic effects due to an imbalance between ROS production and antioxidant defenses [18,19]. Our study observed elevated levels of ROS following MT supplementation, as well as increased activities of SOD, CAT, and POD (Figure 3a,b). Based on these findings, it was observed that an excessive accumulation of ROS led to the activation of oxidative stress, promoting the upregulation of SOD, CAT, and POD to mitigate its effects. Additionally, exposure to MT was found to induce oxidative stress by increasing ROS levels and enhancing activities of SOD and CAT in both zebrafish and rats [18,19]. Several antioxidant-related genes, including fatty acyl-CoA reductase genes (*KWMTBOMO14222*, *KWMTBOMO14221*, *KWMTBOMO14199*, *KWMTBOMO14197*, and *KWMTBOMO14193*) and long-chain-fatty- acid-CoA ligase 4 gene (*KWMTBOMO02325*), were found to be upregulated in the presence of MT, as illustrated in Figure 4, Figure 5a, and Figure S1. These upregulated genes may serve as antioxidants to mitigate the effects of oxidative stress in silkworm larvae by removing ROS.

**Figure 8:**
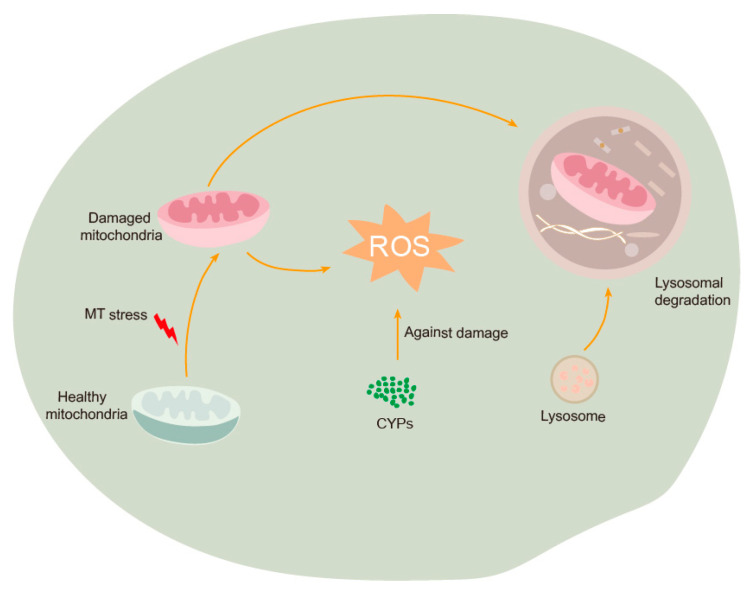
Scheme of CYP responses to MT stress in silkworm midgut.

Glutathione transferases (GST) are widely distributed enzymes capable of catalyzing the conjugation of glutathione (GSH) with a variety of molecules to combat oxidative stress [25,33]. In our investigation, six genes encoding GST exhibited significant alterations (Figure S1), potentially indicating that exposure to MT stress may lead to the accumulation of toxic compounds in silkworms. The activation of GST enzymes under such conditions may facilitate the conversation of endogenous harmful substances generated by MT stress into nontoxic derivatives that can be readily eliminated or degraded. Similar findings were noted in *Helicoverpa armigera*, *Tribolium castanum*, and *Myzus persicase,* where the activity of GST was enhanced under ultraviolet-B light exposure. Furthermore, it was observed that ultraviolet-B radiation induced the accumulation of ROS, leading to the upregulation of expression [34,35,36]. CYPs constitute a group of enzymes capable of metabolizing a diverse array of endogenous and exogenous compounds to safeguard living organisms, with metabolic detoxification representing a crucial function of this enzyme system [37,38,39]. In our investigation, we observed the induction of multiple CYP genes (Figure 6b), suggesting a potential role for these enzymes in both detoxification processes and antioxidant defense mechanisms against ROS damage (Figure 8).

Additionally, we identified the activation of several genes related to lysosome pathway in our study (Figure 6c). Lysosomes play a crucial role as central signaling hubs in various cellular processes, including cellular homeostasis, tumorigenesis, immune responses, and development [40,41,42]. They receive and break down materials obtained through the endocytosis of small molecules and cell surface proteins, as well as the phagocytosis of large particles [40]. Indeed, our TEM results indicate that MT elicited an autophagic response in larvae midgut (Figure 3b). Furthermore, MT stress resulted in disordered mitochondrial morphology and stretched mitochondrial inner membranes (Figure 3b). Therefore, it is reasonable to hypothesize that mitochondria damaged by MT are subsequently degraded via the lysosomal pathway (Figure 8).

Furthermore, analysis using the KEGG revealed that silkworms fed on mulberry leaves containing 10 mg/mL of MT exhibited a significant upregulation of DEGs related to amino acid metabolic processes, specifically arginine, phenylalanine, histidine, and tyrosine metabolism in eukaryotes (Figure 5b and Figure 6d). These findings suggest that the impact of MT on silkworm larval development primarily involves the regulation of amino acid metabolic pathways. It is noteworthy that all DEGs associated with protein export were downregulated, whereas the majority of DEGs (10 out of 11) involved in amino acid biosynthesis were upregulated (Figure 6d,e). This suggests that MT stress may serve as a signal for silkworm larvae to initiate amino acid biosynthesis in order to produce proteins necessary for growth, survival, and defense against MT toxicity. Conversely, the inhibition of protein export may contribute to the toxic effect of MT on larval growth. The ability of insects to tolerate environmental stress is closely linked to metabolic processes that regulate energy balance. Hence, it is suggested that the impact of MT on the metabolic pathway is mediated by the regulation of genes within the amino acid biosynthesis pathway. Nevertheless, additional research is warranted to elucidate the underlying mechanism in subsequent studies. Future research will focus on investigating the role of CYPs (BmorCYP4C1, BmorCYP6B1, and BmorCYP4C3) in silkworm detoxification and resistance against oxidative stress induced by MT.

## 5. Conclusions

In conclusion, this study revealed that exposure to MT resulted in reduced larval weight and induced oxidative stress and autophagy in silkworms. RNA-seq analysis demonstrated that MT primarily impacted the antioxidant, detoxification, lysosome biogenesis, and metabolic pathway in the larvae. These findings elucidate the molecular mechanisms underlying MT induction and offer valuable insights for the potential utilization of MT in mulberry gardens.

## Figures and Tables

**Table 1:** Summary of transcriptome sequencing data.

Samples	Control_1	Control_2	Control_3	MT_10 1	MT_10 2	MT_10 3
Clean reads	41,628,434	38,167,832	40,405,632	38,790,212	39,364,340	43,447,812
Clean bases	6,244,265,100	5,725,174,800	6,060,844,800	5,818,531,800	5,904,651,000	6,517,171,800
Q30 of clean reads	93.35%	93.18%	93.88%	93.36%	95.70%	93.66%
GC contents	47.54%	46.69%	45.93%	46.69%	46.13%	46.68%
Total mapped reads	37,862,142 (90.95%)	28,509,563 (74.70%)	35,610,569 (88.13%)	35,061,892 (90.39%)	36,449,260 (92.59%)	39,820,870 (91.65%)
Uniquely mapped reads	33,216,284 (87.73%)	25,060,252 (87.90%)	31,468,498 (88.37%)	32,053,845 (91.42%)	33,896,516 (93.00%)	36,639,623 (92.01%)
Multiple mapped reads	4,645,858 (12.27%)	3,449,311 (12.10%)	4,142,071 (11.63%)	3,008,347 (8.58%)	2,552,744 (7.00%)	3,118,347 (7.99%)

## Data Availability

The RNA sequence raw data were deposited in the China National Center for Bioinformation (CNCB) Genome Sequence Archive (GSA; accession number CRA009488).

## Supplementary Materials

The following supporting information can be downloaded at: https://www.mdpi.com/article/10.3390/genes15101279/s1, Figure S1: Multiple sequence alignment of silkworm BmorCYP4C1 (KWMTBOMO15838) protein and homologous sequence. Figure S2: Multiple sequence alignment of silkworm BmorCYP4C3 (KWMTBOMO01330) protein and homologous sequence. Figure S3: Multiple sequence alignment of silkworm BmorCYP4B1 (KWMTBOMO12722) protein and homologous sequence. Table S1: Gene-specific primers used for RT-qPCR analysis. Table S2: Differentially expressed genes in *Bombyx mori* larvae induced by methyl-thiophanate. Table S3: The 15 most enriched KEGG pathway analysis of upregulated genes in *Bombyx mori* larvae induced by methyl-thiophonate. Table S4: The 15 most enriched KEGG pathway analysis of downregulated genes in *Bombyx mori* larvae induced by methyl-thiophonate. File S1: BmorCYP4C1 amino acid sequences from different species. File S2: BmorCYP6B1 amino acid sequences from different species. File S3: BmorCYP4C3 amino acid sequences from different species.
