# Supplementary material for: Toxic Effect of Methyl-Thiophanate on Bombyx mori Based on Physiological and Transcriptomic Analysis
Source: Genes (Basel). 2024 Sep 29;15(10):1279. doi: 10.3390/genes15101279 (PMC11507533; doi:10.3390/genes15101279)
Supplement: Supplementary file 1 [file genes-15-01279-s001.zip › FigureS1-S3.pdf]

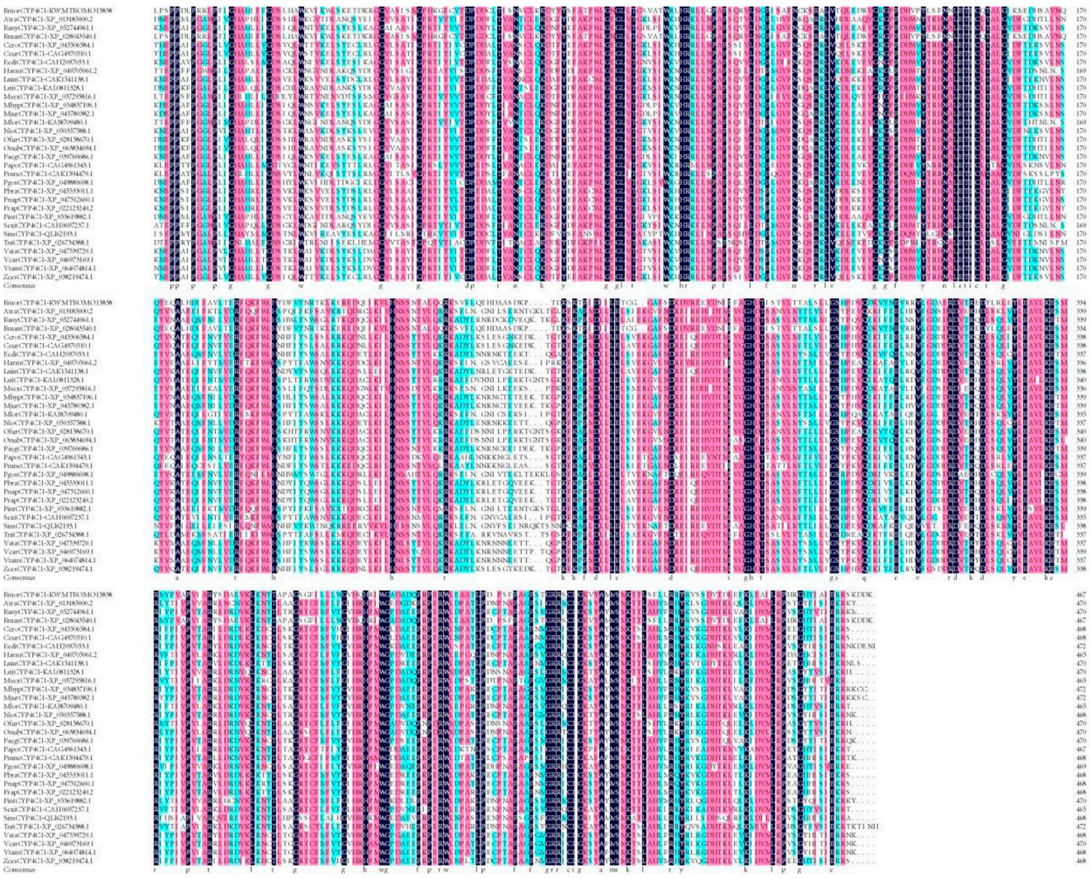

Figure S1 Multiple sequence alignment of silkworm BmorCYP4C1 (KWMTBOMO15838) protein and homologous sequence.

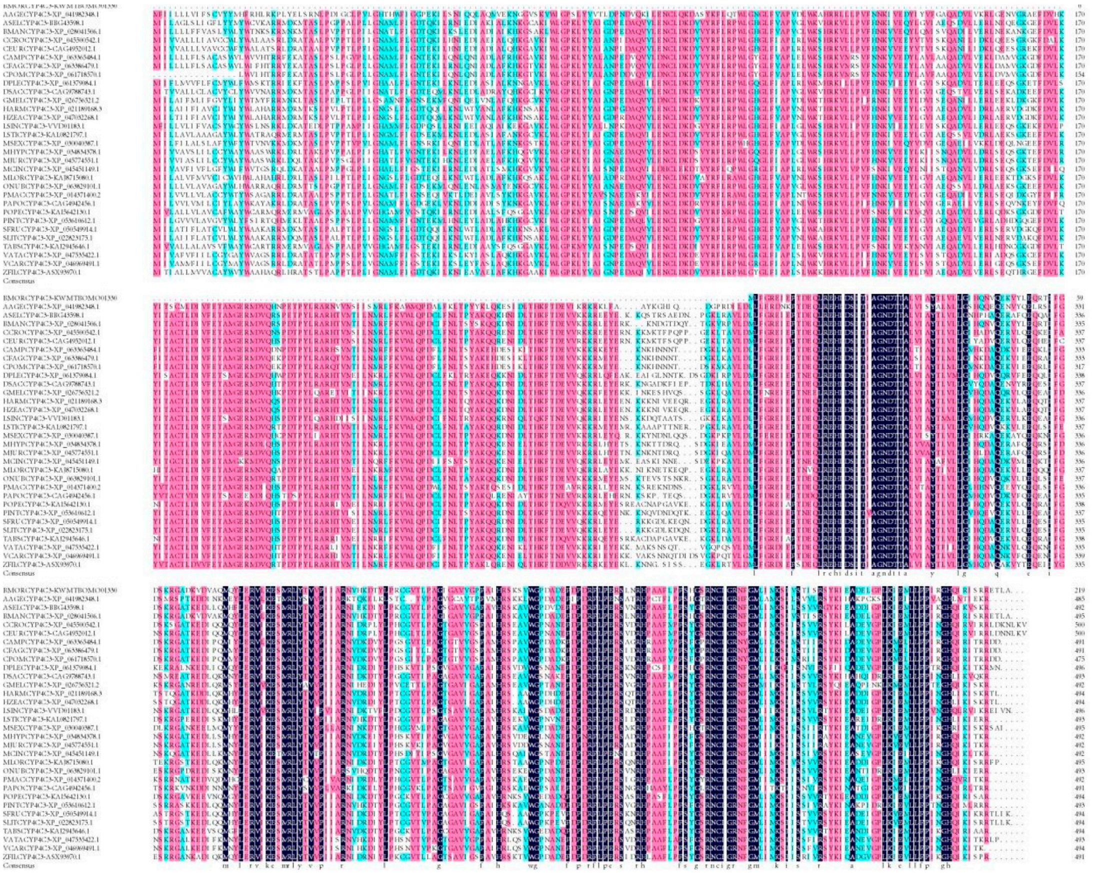

Figure S2 Multiple sequence alignment of silkworm BmorCYP4C3 (KWMTBOMO01330) protein and homologous sequence.

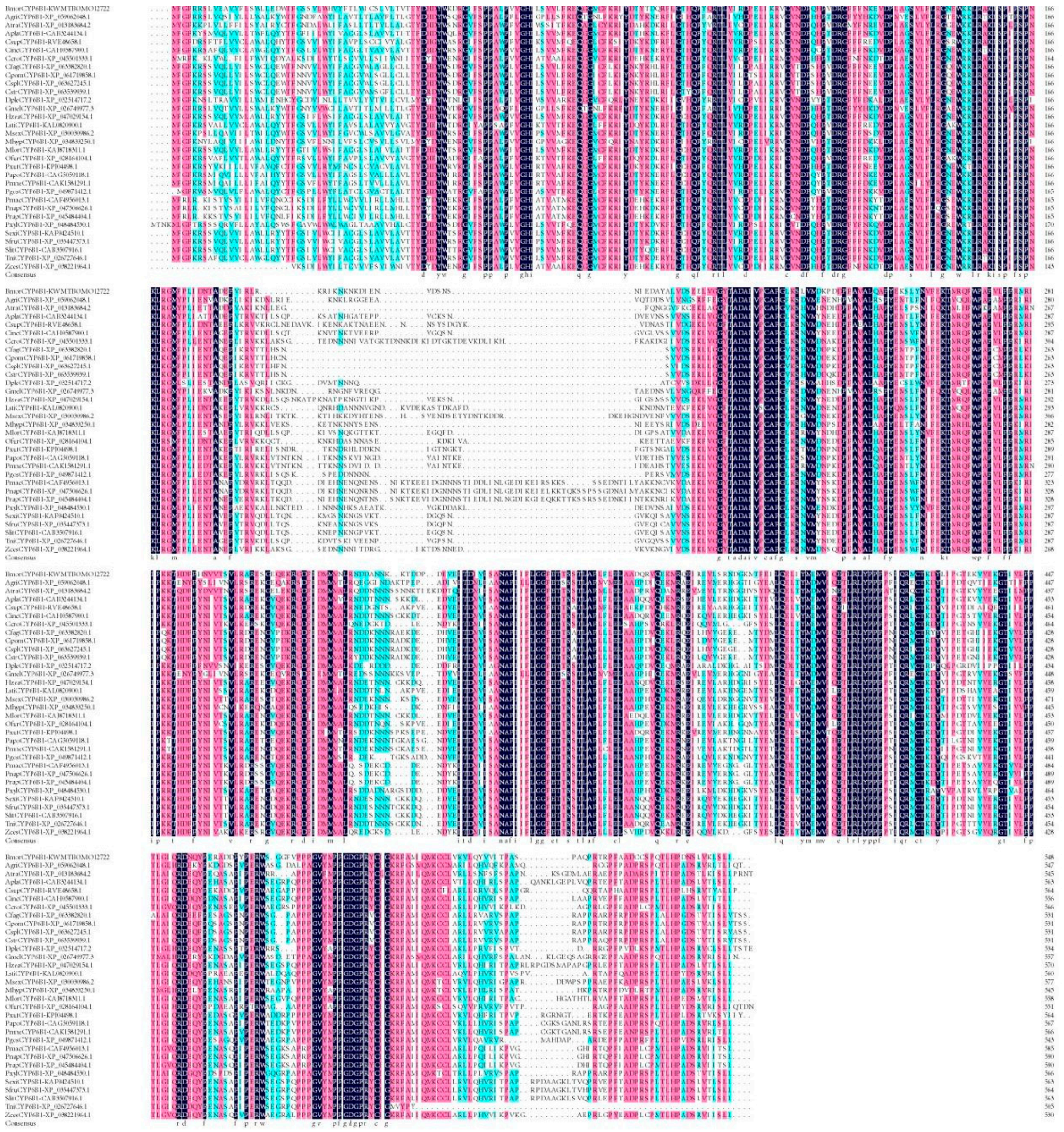

Figure S3 Multiple sequence alignment of silkworm BmorCYP4B1 (KWMTBOMO12722) protein and homologous sequence.
